# Feeding Preferences of the Italian Roe Deer (*Capreolus capreolus italicus* Festa, 1925) in a Coastal Mediterranean Environment

**DOI:** 10.3390/ani11020308

**Published:** 2021-01-26

**Authors:** Pierangelo Freschi, Simonetta Fascetti, Francesco Riga, Gabriella Rizzardini, Mauro Musto, Carlo Cosentino

**Affiliations:** 1 School of Agricultural, Forestry, Food and Environmental Sciences (SAFE), University of Basilicata, 85100 Potenza, Italy; pierangelo.freschi@unibas.it (P.F.); simonetta.fascetti@unibas.it (S.F.); gabriellarizzardini@virgilio.it (G.R.); mauro.musto@gmail.com (M.M.); 2 Italian Institute for Environmental Protection and Research (ISPRA), 00144 Rome, Italy; francesco.riga@isprambiente.it

**Keywords:** Italian roe deer, feeding preferences, diet, micro-histological analysis

## Abstract

**Simple Summary:**

The Italian roe deer, once largely diffused in central and southern Italy, currently populates residual areas of its historical range in an extremely precarious status, with a numerical consistency which has been reduced to a few thousand heads. Moreover, the introgressive hybridization due to the occurrence in close contact with the European roe deer, in some areas of Tuscany and Calabria, threatens the genetic identity of the endemic subspecies. Therefore, the numerous risk factors for this subspecies require enabling targeted conservation strategies. In the present research we studied the diet and feeding behavior of the Italian roe deer, considering that the most representative plants of the diet could act as key indicators for the subspecies to identify and conserve its elective habitats. During all the year, the Italian roe deer preferred mainly feeding resources from woods and scrubland including, particularly in wet season, also a great proportion of forbs, whereas in its diet grasses were poorly represented. The results obtained in this study evidenced that the Italian roe deer is a browser able to exploit many plant species and to adapt its diet preferences to space-time variation of food availability.

**Abstract:**

The present study was aimed to deepen the knowledge on diet and on feeding preferences of the Italian roe deer. The research was carried on in the Castelporziano Presidential Estate, a protected area of Latium coast. Quadrat method was used to assess plant frequency, whereas diet composition was determined by fecal micro-histological analysis. A total of 143 plant species were identified in the site and 109 of them were found in the feces of the *Capreolus capreolus italicus*. All over the year, most of the *taxa* were ingested in low percentages (≤5%) and the most utilized plant species were *Quercus suber*, *Prunus spinosa*, *Rubia peregrina*, and *Crateaegus monogyna*. Fagaceae and Rosaceae plant families represented the bulk of the diet and were positively selected. In addition, a positive selection on Fabaceae and Oleaceae families was found in wet season but not in dry one. In both periods the Italian roe deer showed a positive selection for deciduous woody plants and evergreen woody plants, instead avoided wild forbs and wild graminoids. Our results confirmed that this subspecies behaved as a generalist highly selective feeder.

## 1. Introduction

Studies on feeding ecology and diet composition of a threatened taxon as the Italian roe deer (*Capreolus capreolus italicus* Festa, 1925) are essential to identify potential factors influencing the population viability, as well as for protection of its elective habitats [1] Additionally, plants composing the diet may act as early warning indicators of food resource limitation, especially in relation to diet overlap with other animals, and are essential to assess the species’ role in the ecosystem and in the development of management or reintroduction plans [2].

The Italian roe deer is currently confined in only a few areas: Sienese hills and Maremma Regional Park (Tuscany), Castelporziano Presidential Estate (Latium), Umbra Forest (Gargano Regional Park, Apulia), Orsomarso mountains (Pollino National Park, Calabria). Thanks to recent reintroductions, it is also present in the Gallipoli Cognato Piccole Dolomiti Lucane Regional Park (Basilicata) and in the Aspromonte National Park (Calabria) [1]. The Italian roe deer occupies a diversity of habitat, mainly deciduous forests of the Southern Apennines, Mediterranean scrublands and agricultural areas. The distinction of this endemism from the European roe deer (*Capreolus capreolus* Linnaeus, 1758) was suggested for the first time by Festa (1925). Later, the Italian roe deer was genetically differentiated from the nominal species [2,3,4]. Only the populations of Castelporziano and of Umbra Forest, confined in two protected areas and isolated from the European roe deer, are not threatened by hybridization [5,6].

Ecological studies on the Italian roe deer mainly concern the spatial behavior and habitat use of both historical and southern Tuscany populations [7,8,9,10,11,12], only little is known about its feeding ecology. The few studies focused on feeding behavior of this small cervid [12,13,14] put in evidence that its diet is wide and diversified with only a few plant species ingested in higher percentage. Studies on the diet of roe deer regard mostly the European roe deer and are mainly localized in forest areas of central and northern Europe e.g., [15,16,17,18,19]. These researches evidenced a wide selection of plant species (about 1000 at a continental scale) with high-energy content and high digestibility (especially herbaceous dicotyledons, followed by woody plants and monocotyledons). In general, roe deer is defined as a concentrate selector, and its ecological plasticity is highlighted by its adaptation in response to the different availability of feeding resources [13] and to the vegetation phenology, since roe deer selects plants in the most nutritive phenological stages [20].

Nevertheless, information obtained from these studies might prove poorly appropriate for the Italian roe deer, predominantly distributed in Mediterranean habitats [2,12].

The present research was carried on in a protected area of the Latium coast with the aim of studying the seasonal variations of diet and feeding selection behavior of the Italian roe deer.

## 2. Materials and Methods

### 2.1. Study Site

**Figure 1:**
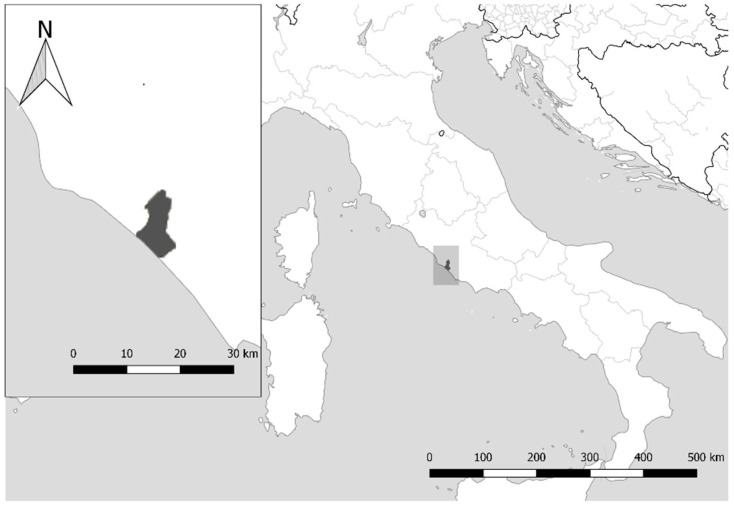
Map showing the study site in Latium coast.

The study was conducted in the Castelporziano Presidential Estate (Figure 1), an enclosed and protected area of Lazio coast (headquarters coordinates: 41°44′37.83″ N, 12°24′2.20″ E) where the mean annual temperature is +15.4 °C and the annual precipitation is 740 mm. The Estate covers about 5892 ha containing several land-cover types representative for the Mediterranean area: natural oak woods with both evergreen (*Quercus ilex* and *Quercus suber*) and deciduous (*Quercus cerris* and *Quercus frainetto*) species, broad-leaved mixed oak forest, pasture, Mediterranean maquis, pseudo steppe and reforestation areas with prevalence of domestic pine (*Pinus pinea*) [21].

A sampling site of about 400 ha was chosen in the north of the Estate in order to include different vegetational covers. Ground cover of this area is characterized by: (1) a dominant tree layer of *Pinus pinea*, by sparse shrub undergrowth with *Asparagus acutifolius*, *Laurus nobilis*, *Phillyrea latifolia*, and *Rubus* spp. and a very scarce herbaceous layer formed by *Carex dystachya*, *Carex flacca* and *Poa trivialis*; (2) a tree pasture area, with scattered specimens of *Quercus suber*, with prevailing annual-growing grasses as herbaceous species (e.g., *Anthoxanthum odoratum*, *Briza maxima*, *Bromus hordeaceus* and *Cynosurus echinatus* and *Trifolium* spp.), the presence of nitrophilous spiny species, such as *Cirsium strictum* and *Galactites tomentosa*, is probably affected by cattle overgrazing [22]; (3) a fallow area characterized by annual growing grasses (in prevalence, *Avena fatua*, *Cynodon dactylon*, *Dasypyrum villosum*, *Lagurus ovatus* and *Poa trivialis*). The control activity of the estate gamekeepers excludes from the study area the presence of fallow deer (*Dama dama* Linnaeus, 1758) and wild boar (*Sus scrofa majori* De Beaux e Festa, 1927), species that may interfere on feeding behavior of the Italian roe deer [8,23]. The site is not usually frequented by Red deer (*Cervus elaphus hippelaphus* Erxleben, 1777).

### 2.2. Sampling and Analysis Procedures

For each vegetational cover, two permanent transects were performed and sampling took place once in dry season (DS, May–August) and once in wet season (WS, November–February). Transects were distant from each other at least 100 m. Quadrat method was used to assess plant frequency [24]: in each transect (100 m) 50 samplings were made analyzing 1 m^2^ of vegetation and skipping the following. Identified species were grouped into five vegetation forms: evergreen woody plants (EWP), deciduous woody plants (DWP), half-woody plants (HWP), wild forbs (WF) and wild graminoids (WG). The taxonomic nomenclature of the identified taxa follows Bartolucci et al. [25]. Sampling of plants species took place along the above descripted transects to create a reference collection. The plant material was divided into anatomical parts and crushed in a ceramic mortar. Fragments were dispersed in a few drops of water, placed on microscope slides and put in oven at 50 °C for 30′. Histological fragments were photographed by light microscopy and catalogued in a database using the image analyzer Leica Q500IW (Leica Imaging System Ltd., Cambridge, UK).

Fecal sampling took place in DS and WS along 3 replicate and permanent transects (2 m × 200 m), spatially distributed in order to include the different vegetation covers of the site.

Pellets were collected monthly in DS (June–August) and in WS (November–February). From each collection a minimum of 6 fresh pellets (bright brown feces) of various sizes and formats, were mixed to form a single composite sample. Overall, 24 composite samples (8 months × 3 sites) were analyzed.

Fecal pellets were hydrated, homogenized and placed in sodium hypochlorite (NaClO) for 4 h, in order to allow the discoloration of plant fragments Successively, fragments were washed with water and collected with 400 μm filter paper [26]. The filtrate was dried at 50 °C for 90′ and mounted in glycerol gelatin on microscope slides. For each composite sample were mounted 10 microscope slides. The slides were examined by light microscopy by using the image analyzer Leica Q500 IW (Leica Imaging System Ltd., Cambridge, UK), obtaining 200 readings for each sample, counting non-overlapping plant fragments in systematic transects across a slide along alternate rows.

Identification of plant species was affected by comparing the different characteristic of the epidermal cells and other structures (e.g., stomates and trichomes) with those of the plant reference collection. This reference material is available at the Laboratory of Environmental and Applied Botany, University of Basilicata. Not identified fragments (5.4%) were classified as ‘unidentified’ and excluded from the analysis.

### 2.3. Statistical Analysis

Data on the plant species identified in DS and WS were used to calculate the relative frequencies of each taxon, family, and vegetation form. Similarly, we calculated the relative frequencies of the plant species identified in the feces by dividing the total number of fragments attributed to a given taxon by the total number of identified fragments collected for each season [27,28,29]. Data on identified plant species composing the diet were also used to compute the following alpha diversity indices:

- Shannon diversity index (*H*) [30], whose value usually ranges between 1.5 and 3.5 and often does not exceed 4 [31];
- Margalef index (*D*) for species richness (higher the value the greater is the richness) [32];
- Buzas and Gibson evenness index (*E*) [33].

For each of the above indices’ differences between DS and WS were tested by Student’s *t*-test.

To compare dietary similarity between DS and WS two indices were computed: the Sørensen similarity index (*C_S_*) [34] and the Morisita-Horn index (*C_M_*) [35]. Both indices vary between 0 (no similarity) and 1 (complete similarity). Morisita-Horn index values were classified as: 0 < *C_M_* ≤ 0.29 small overlap, 0.30 ≤ *C_M_* ≤ 0.59 medium overlap, and *C_M_* ≥ 0.60 high overlap [36].

Diet selection was estimated for life vegetation forms and for plant families, utilizing the relative frequencies, in vegetation and diet by Resource selection ratio (*w_i_*) [37]:

*w_i_* = *o_i_/p_i_* (1)

where *o_i_* is the proportion of the botanical family (or life form) in the diet and *p_i_* is its available proportion. Differences were tested by *χ*^2^ test [38]. Data were analyzed by R software [39].

## 3. Results

### 3.1. Vegetation Assessment of the Site

Relative frequencies of each *taxon*, family, and vegetation form composing vegetation cover are shown in Table A1. Overall, 143 plant species were detected (72 in DS and 71 in WS), belonging to 56 families (26 in DS and 30 WS). The most represented families were Poaceae (49.1% in DS and 19.8% in WS), Asteraceae (20.8% in DS and 16.3% in WS), Rosaceae (9.2% in DS) and Geraniaceae (9.5% in WS). Among inventoried species the most representative ones were *Quercus suber* (8.9%), *Avena fatua* (6.6%), *Dasypyrum villosum* (6.2%), *Achnatherum bromoides* (6.1%), and *Briza maxima* (5.5%) in DS and *Quercus suber* (19.4%), *Rubus ulmifolius* (5.6%), *Geranium robertianum* (5.4%), *Brachypodium retusum* (5.0%) and *Dactylis glomerata* (4.5%) in WS.

During all the year, the most abundant vegetation forms were wild forbs (48.7%) followed by wild graminoids (33.6%), evergreen woody plants (10.0%), deciduous woody plants (7.0%) and half-woody plants that was the less abundant form (0.8%).

### 3.2. Diet Composition

A total of 109 *taxa* belonging to 51 families was found in the feces of *Capreolus capreolus italicus* (Table A1). The number of identified species/families was similar in both seasons (DS: 56/29; WS: 53/22). Among plant species the most ingested were *Quercus. suber* (13.3%), *Prunus spinosa* (9.0%), *Rubia peregrina* (7.7%) and *Crateaegus monogyna* (5.4%). In DS the most consumed species were: *Rubia peregrina* (10.11%), *Quercus suber* (8.9%) and *Osyris alba* (7.51%). In WS the most utilized were: *Quercus suber* (19.4%), *Prunus spinosa* (15.2%), *Crataegus monogyna* (9.2%) and *Pyrus communis* (7.8%). Overall, in the two periods, most of the *taxa* were ingested in low percentages (≤5%), giving 64.5% of total.

All over the year, Rosaceae was the most representative family in the diet (20.8%), followed by Fagaceae (17.0%) and Rubiaceae (10.7%). In DS the family of Rubiaceae was the most ingested (14.6%), followed by Fagaceae (14.4%) and Rosaceae (11.6%) while in WS the most representative families in the diet were Rosaceae (33.4%), Fagaceae (20.6%) and Poaceae (15.7%).

### 3.3. Seasonal Variation in Dietary Diversity and Similarity

Table 1 provides the results obtained by computing the alpha and beta diversity indices in the two seasons. The Student’s *t*-test revealed significant differences in terms of diet diversity. The value of the Shannon index was significantly higher (*t* = 4.733, df = 4.999, *p* ≤ 0.01) in WS than in DS (3.312 vs. 2.490). Similarly, we also found a significant difference concerning diet richness: the value of the Margalef’s index was significantly higher (*t* = 6.583, df = 4.668, *p* ≤ 0.01) in WS compared to DS (7.415 vs. 2.490). No significant difference was found when comparing the value of Buzas and Gibson’s index (*t* = 1.766, df = 4.879, *p* = 0.14).

Concerning beta diversity analysis, the DS and WS diets showed a relative low value of *C_S_* (0.28). However, observing the value of the Morisita-Horn index, the degree of dietary overlap can be defined “high” (*C_M_* = 0.76).

### 3.4. Dietary Selection

**Figure 2:**
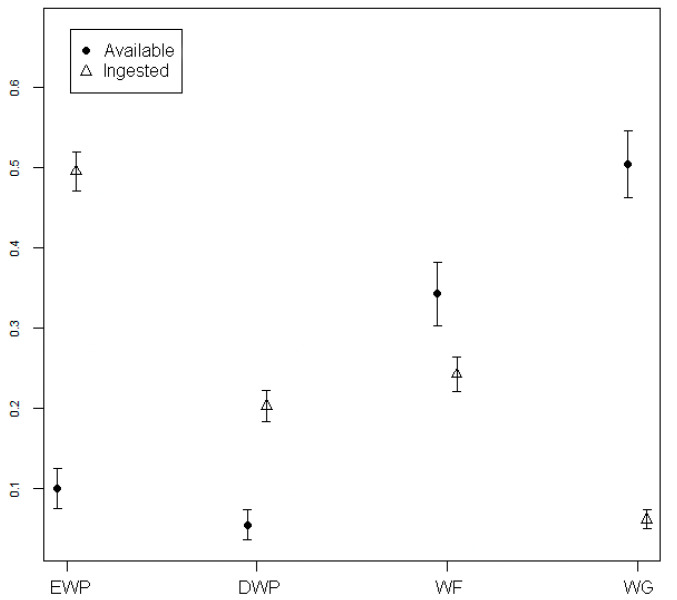
Ingested and available plants grouped by vegetation form in DS (EWP = Evergreen woody plants; DWP = Deciduous woody plants; WF = Wild forbs; WG = Wild graminoids).

**Figure 3:**
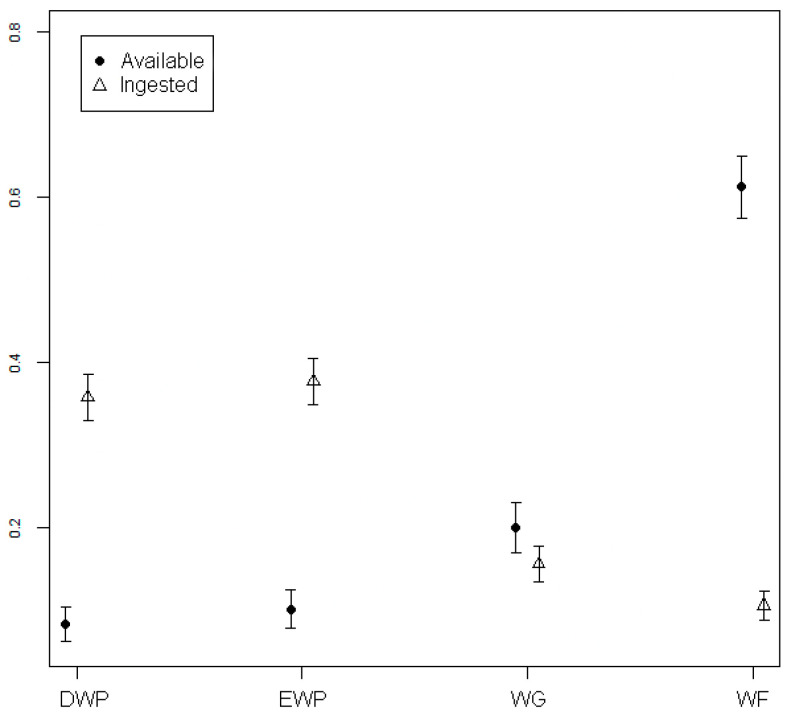
Ingested and available plants grouped by vegetation form in WS (EWP = Evergreen woody plants; DWP = Deciduous woody plants; WF = Wild forbs; WG = Wild graminoids).

The Italian roe deer showed a positive selection in DS and WS for deciduous woody plants and evergreen woody plants (*p* ≤ 0.001); wild forbs and wild graminoids were instead avoided (*p* ≤ 0.001) (Figure 2 and Figure 3).

In Table 2 is reported the selection ratio (*w_i_*) of the Italian roe deer on botanical families. In DS, Fabaceae, Fagaceae, Oleaceae and Rosaceae families have been used more than expected according to their availability (*p* ≤ 0.05); conversely, Apiaceae, Asteraceae, Poaceae and Smilacaceae were negatively selected (*p* ≤ 0.001). In WS only Fagaceae and Rosaceae were positively selected (*p* ≤ 0.05), whereas Apiaceae, Asteraceae, Fabaceae, Plantaginaceae and Poaceae were avoided (*p* ≤ 0.001).

## 4. Discussion

Four main results emerged from our study: (1) annual diet composition was characterized by a broad spectrum of plant species, around 110 belonging to 12 families; (2) use and selection of food was conditioned not only by the seasonal availability of plant species but also by their phenological stage, e.g., Fabaceae were preferred in DS and avoided in WS; (3) all over the year, *Quercus suber*, *Prunus spinosa*, *Rubia peregrina* and *Crataegus monogyna* represented the bulk of diet; these species were observed in the diet of the European roe deer too [11,18,40,41]; (4) comparing alpha and beta indices, we found that in wet season the diet was more diverse and richer than in dry season, and that there was an even distribution of plant species eaten in each season; besides, some of these species were shared by the diets, and this was particularly evident if taking species abundance into consideration.

During all the year, Fagaceae and Rosaceae represented the bulk of the diet, and were positively selected. In addition, in summer we found a positive selection on Fabaceae and Oleaceae families as well. These results seem to support the findings by Focardi et al. [23] who suggested that the availability of high-quality food resources makes woods and scrubland the most preferred habitats by the Italian roe deer.

The selection on Rosaceae has been highlighted by the identification of fruits in the feces and confirms that these cervids seek out more palatable and high-quality food sources. Previous studies have shown that fruits are the most preferred food items in summer and in early autumn for the European roe deer [42]. In a study on summer diet of the subspecies [14], Rosaceae and Fagaceae represented the bulk of the diet in both a coastal and mountain environment.

Mammalian herbivores can be considered generalist or specialist in feeding behavior if the incidence of a family plant on the diet is less than or greater than 60%, respectively [43]. In this study the generalist behavior in the Italian roe deer was confirmed. At the same time, it was also evidenced the high selectivity of this endemism: if we consider the 4 most abundant species in the diets of the two periods they accounted for 35.4% in WS and 27.9% in DS.

Despite the large diet breath, diet selection occurred, because deciduous woody plants and evergreen woody plants were selected in both periods, whereas wild forbs and wild graminoids were avoided. Evergreen woody plants represented the basic diet for the Italian roe deer (45%), in agreement with previous studies in other areas: up to 65% in Maremma Regional Park [13] and over 50% in the province of Siena, where evergreen woody plants were selected in autumn-winter when the availability of deciduous plants is reduced [12]. Deciduous woody plants were used more than expected by their availability, in particular in wet season. In literature, this biological form is selected and utilized in similar amounts by the European roe deer [13].

The relatively low proportion of ingested wild forb *taxa* may be attributed to their better digestibility that can give a biased idea of the actually utilized species. Studies on diet selection show considerable variation with respect to spatial and temporal scales and methods employed to measure resource use and availability [15,44,45]. Conclusions about whether a single vegetation form is used above, in proportion to, or below its availability are directly dependent upon the accuracy of diet and availability assessments, but also upon which categories are deemed available [44]. However, avoidance of many forbs and graminoids and use of almost all tree and shrub species were confirmed by other studies based on browsing marks and direct observation of the European roe deer [46]. The results obtained in this study are in accordance with literature regarding the food preferences of the European roe deer that is commonly recognized as a browser, capable of exploiting a large number of plant species and adapting its dietary niche to the space-time variation of food availability [17,47,48,49]. The Italian roe deer managed to model its feeding behavior in relation to the available food resources in the dry and wet periods, satisfying its metabolic demand with variable proportions of different plant categories.

## 5. Conclusions

Our results showed that, although the Italian roe deer heavily relied on woody plants, its diet was quite richer and diverse all year long, due to the availability and the phenological stage of the plants. These results confirm that feeding behavior of the Italian roe deer is plastic, changing with the seasonal availability of feeding resources [17,20]. Knowledge of the diet and of the feeding behavior of herbivorous species is an important element for the definition of their trophic niche, of their elective habitats, and of the competition with other taxa [50]. Our results could give useful indications for the management of this subspecies in similar environments of the Mediterranean area. Nevertheless, studies conducted at multiple scales [20] could, provide a fuller characterization of habitat use patterns and a far-reaching impact on the development of reintroduction plans.

## Figures and Tables

**Table 1:** Diet of *Capreolus capreolus italicus* in dry season (DS) and in wet season (WS): diversity and similarity indices.

Index	DS		WS		*p*-Value
	Mean	SE	Mean	SE	
Diversity					
Shannon, *H*	2.490 ± 0.064		3.312 ± 0.045		<0.01
Margalef, *D*	4.589 ± 0.173		7.415 ± 0.091		<0.01
Buzas & Gibson, *E*	0.442 ± 0.027		0.564 ± 0.016		0.14
Similarity					
Sørensen, *C_S_*	0.28				
Morisita-Horn, *C_M_*	0.76				

**Table 2:** Selection ratio (*w_i_*) of *Capreolus capreolus italicus* on botanical families in dry season (DS) and in wet season (WS).

Family	DS			WS		
	*w_i_*	Feeding Behavior	*p*-Value	*w_i_*	Feeding Behavior	*p*-Value
Apiaceae	0.139	A	<0.001	0.021	A	<0.001
Asparagaceae	1.401	I	0.80	1.068	I	0.07
Asteraceae	0.034	A	<0.001	0.032	A	<0.001
Cistaceae	14.824	I	0.93	2.757	I	0.62
Fabaceae	2.713	P	0.05	0.365	A	<0.001
Fagaceae	9.893	P	<0.001	25.888	P	0.05
Oleaceae	5.009	P	<0.01	0.793	I	0.62
Plantaginaceae	2.118	I	0.18	0.052	A	<0.001
Poaceae	0.052	A	<0.001	0.562	A	<0.001
Rosaceae	3.344	P	<0.001	2.621	P	<0.001
Rubiaceae	160.412	I	0.13	0.985	I	0.15
Smilacaceae	0.265	A	<0.001	16.128	I	0.24

Feeding behavior: (P) preference, (I) indifference, (A) avoidance.

## Data Availability

Plant reference collection is available at the Laboratory of Environmental and Applied Botany, University of Basilicata, Potenza, Italy.

## Appendix A

Table A1: Frequencies (%) of Plant species, Families, and Vegetation form (1) in dry season (DS) and in wet season (WS), in vegetation (availability) and in diet (ingested).

**Table A1:** Frequencies (%) of Plant species, Families, and Vegetation forms (^1^) in dry season (DS) and in wet season (WS), in vegetation (availability) and in diet (ingested).

Family	Plant Species	Vegetation Form	DS		WS	
			Availability	Ingested	Availability	Ingested
Amaryllidaceae	*Allium triquetrum*	WF	0.83	0.00	2.61	0.20
			0.83	0.00	2.61	0.20
Anacardiaceae	*Pistacia lentiscus*	EWP	0.00	0.77	0.00	0.00
			0.00	0.77	0.00	0.00
Apiaceae	*Chaerophyllum* spp.	WF	0.00	0.00	2.61	0.00
	*Daucus carota*	WF	2.22	0.00	0.23	0.00
	*Foeniculum vulgare*	WF	0.28	0.24	0.00	0.07
	*Oenanthe pimpinelloides*	WF	0.00	0.00	1.59	0.07
	*Smyrnium olusatrum*	WF	0.14	0.00	0.00	0.00
			2.64	0.24	4.42	0.13
Araceae	*Arum italicum*	WF	0.00	0.00	0.34	0.00
			0.00	0.00	0.34	0.00
Araliaceae	*Hedera helix*	EWP	0.97	1.15	0.00	0.00
			0.97	1.15	0.00	0.00
Asparagaceae	*Asparagus acutifolius*	EWP	2.36	1.49	1.13	1.91
	*Ruscus aculeatus*	EWP	0.00	0.67	0.23	0.13
			2.36	2.17	1.36	2.04
Asphodelaceae	*Asphodelus ramosus*	WF	0.00	0.00	2.27	0.00
			0.00	0.00	2.27	0.00
Asteraceae	*Anthemis cotula*	WF	0.14	0.00	3.97	0.00
	*Bellis perennis*	WF	0.00	0.00	3.17	0.00
	*Carlina corymbosa*	WF	0.00	0.00	0.34	0.00
	*Carthamus lanatus*	WF	0.28	0.00	0.00	0.00
	*Centaurea solstitialis*	WF	5.41	0.00	0.00	0.00
	*Cichorium intybus*	WF	3.05	0.00	0.00	0.00
	*Cirsium arvense*	WF	0.00	0.00	0.11	0.07
	*Cirsium strictum*	WF	2.22	0.00	3.17	0.00
	*Coleostephus myconis*	WF	2.77	0.00	1.02	0.00
	*Dittrichia viscosa*	WF	0.00	0.00	0.23	0.00
	*Erigeronbonariensis*	WF	0.00	0.00	0.34	0.13
	*Erigeron* spp.	WF	0.14	0.00	0.00	0.00
	*Galactites tomentosa*	WF	3.19	0.00	0.00	0.00
	*Helminthothec aechioides*	WF	2.77	0.00	0.00	0.00
	*Hypochaeris radicata*	WF	0.00	0.00	0.00	0.13
	*Hypocheris achyrophorus*	WF	0.00	0.00	0.23	0.00
	*Onopordon illyricum*	WF	0.55	0.00	0.11	0.00
	*Picris hieracioides*	WF	0.83	0.00	0.00	0.00
	*Ptilostemon strictus*	WF	0.00	0.00	0.00	0.26
	*Reichardia picroides*	WF	0.00	0.00	3.63	0.00
	*Rhagadiolus stellatus*	WF	0.14	0.00	0.00	0.00
	*Sonchus oleraceus*	WF	0.00	0.00	0.00	0.13
	*Taraxacum officinale*	WF	0.00	0.48	0.00	0.00
			21.50	0.48	16.33	0.73
Betulaceae	*Alnus glutinosa*	DWP	0.00	2.36	0.00	0.00
			0.00	2.36	0.00	0.00
Boraginaceae	*Cynoglossum* spp.	WF	0.14	0.00	0.00	0.00
	*Echium vulgare*	WF	0.14	0.00	0.00	0.00
	*Myosotis* spp.	WF	0.14	0.00	0.00	0.00
			0.42	0.00	0.00	0.00
Brassicaceae	*Raphanus raphanistrum*	WF	0.14	0.00	0.91	0.00
			0.14	0.00	0.91	0.00
Caprifoliaceae	*Lonicera etrusca*	DWP	0.00	0.00	0.00	0.07
			0.00	0.00	0.00	0.07
Caryophyllaceae	*Silene alba*	WF	0.14	0.00	0.57	0.00
	*Silene colorata*	WF	0.28	0.00	0.00	0.00
	*Stellaria media*	WF	0.00	0.00	2.49	0.00
			0.42	0.00	3.06	0.00
Celastraceae	*Euonymus europaeus*	EWP	0.00	1.20	0.00	0.00
	*Euonymus latifolius*	DWP	0.00	0.00	0.00	0.13
			0.00	1.20	0.00	0.13
Chenopodiaceae	*Chenopodium album*	WF	0.42	0.00	0.00	0.00
			0.42	0.00	0.00	0.00
Cistaceae	*Cistus creticus*	EWP	0.28	0.00	0.68	2.64
	*Cistus monspeliensis*	EWP	0.00	0.67	0.00	0.00
	*Cistus salviifolius*	EWP	0.00	2.02	0.00	0.00
			0.28	2.69	0.68	2.64
Corylaceae	*Corylus avellana*	DWP	0.00	0.63	0.00	0.00
			0.00	0.63	0.00	0.00
Cyperaceae	*Carex echinata*	WG	0.28	0.00	0.00	0.00
	*Carex flacca*	WG	0.69	3.80	0.00	0.00
	*Carex hallerana*	WG	0.28	0.00	0.00	0.00
			1.25	3.80	0.00	0.00
Dioscoreaceae	*Tamus communis*	EWP	0.00	5.39	0.00	0.00
			0.00	5.39	0.00	0.00
Ericaceae	*Arbutus unedo*	EWP	0.00	5.05	0.00	0.00
	*Erica arborea*	EWP	0.00	0.91	0.00	0.00
			0.00	5.97	0.00	0.00
Euphorbiaceae	*Euhorbia helioscopia*	WF	0.28	0.00	0.00	0.00
	*Euphorbia peplis*	WF	0.00	0.00	3.40	0.00
			0.28	0.00	3.40	0.00
Fabaceae	*Astragalus glycyphyllus*	WF	0.00	0.00	0.23	0.00
	*Coronilla scorpioides*	WF	0.14	0.00	0.00	0.00
	*Cytisus scoparius*	EWP	0.00	1.44	0.00	0.00
	*Hippocrepis biflora*	WF	0.00	0.00	0.00	0.07
	*Hippocrepis unisiliquosa*	WF	0.00	0.00	0.57	0.00
	*Lathyrus sylvestris*	WF	0.00	0.00	0.00	0.46
	*Medicago arabica*	WF	0.00	0.00	0.11	0.07
	*Spartium junceum*	EWP	0.00	0.91	0.00	0.00
	*Trifolium alexandrinum*	WF	0.00	0.58	0.00	0.00
	*Trifoliuman gustifolium*	WF	0.14	0.00	0.68	0.00
	*Trifolium pratense*	WF	1.39	0.53	0.23	0.33
	*Trifolium repens*	WF	0.00	0.48	0.00	0.00
	*Trifolium* spp.	WF	0.00	0.00	0.11	0.00
	*Trifolium stellatum*	WF	0.28	0.00	0.00	0.07
	*Trifolium vesiculosum*	WF	0.28	0.00	0.00	0.00
			2.22	3.95	1.93	0.99
Fagaceae	*Quercus cerris*	DWP	0.00	0.00	0.00	0.99
	*Quercus frainetto*	DWP	0.00	4.72	0.00	0.00
	*Quercus ilex*	EWP	0.00	0.77	0.00	0.26
	*Quercus pubescens*	DWP	0.14	0.00	0.23	0.00
	*Quercus suber*	EWP	2.08	8.90	0.34	19.38
			2.22	14.39	0.57	20.63
Geraniaceae	*Erodium cicutarium*	WF	0.00	0.00	0.00	0.07
	*Geranium dissectum*	WF	3.05	0.00	1.93	0.46
	*Geranium robertianum*	WF	0.00	0.00	5.44	0.00
	*Geranium rotundifolium*	WF	0.00	0.00	2.15	0.00
			3.05	0.00	9.52	0.53
Hypericaceae	*Hypericum perforatum*	WF	0.00	0.00	0.57	1.52
			0.00	0.00	0.57	1.52
Juncaceae	*Juncus acutus*	WG	0.00	0.58	0.00	0.00
			0.00	0.58	0.00	0.00
Lamiaceae	*Clinopodium vulgare*	HWP	0.00	0.00	1.36	0.00
	*Lamium album*	WF	0.00	0.00	0.34	0.00
	*Prunella volgaris*	WF	0.00	0.00	0.00	0.79
			0.00	0.00	1.70	0.79
Lauraceae	*Laurus nobilis*	EWP	0.00	0.00	0.23	0.00
			0.00	0.00	0.23	0.00
Liliaceae	*Ornithogalum umbellatum*	WF	0.28	0.00	0.00	0.00
			0.28	0.00	0.00	0.00
Malvaceae	*Malva sylvestris*	WF	0.69	0.00	0.00	0.00
			0.69	0.00	0.00	0.00
Oleaceae	*Fraxinus ornus*	DWP	0.00	0.00	0.00	1.19
	*Ligustrum vulgare*	EWP	0.00	0.34	0.00	0.00
	*Olea europea*	EWP	0.00	0.43	0.00	0.00
	*Phyllirea latifolia*	EWP	1.80	5.15	4.08	3.36
			1.80	5.92	4.08	4.55
Oxalidaceae	*Oxalis corniculata*	WF	0.00	0.00	1.13	0.20
			0.00	0.00	1.13	0.20
Pinaceae	*Pinus pinea*	EWP	0.00	0.00	0.45	0.00
			0.00	0.00	0.45	0.00
Plantaginaceae	*Plantago crassifolia*	WF	0.00	0.53	0.00	0.00
	*Plantago lanceolata*	WF	0.42	0.43	2.61	0.13
	*Plantago media*	WF	0.28	0.00	0.11	0.07
			0.69	0.96	2.72	0.20
Poaceae	*Achnatherum bromoides*	WG	6.24	0.00	0.00	0.00
	*Antoxantum odoratum*	WG	0.14	0.00	0.00	0.00
	*Arrhenatherum elatius*	WG	3.61	0.00	0.00	0.00
	*Avena barbata*	WG	0.00	0.00	0.00	1.25
	*Avena fatua*	WG	6.80	0.00	0.00	0.00
	*Brachypodium retusum*	WG	0.00	0.00	4.99	0.07
	*Brachypodium sylvaticum*	WG	0.28	0.58	0.23	2.90
	*Briza maxima*	WG	5.69	0.00	3.06	0.13
	*Bromus hordeaceus*	WG	1.66	0.00	0.00	0.00
	*Cynodon dactylon*	WG	0.28	0.53	0.00	0.13
	*Cynosurus cristatus*	WG	0.00	0.00	1.59	0.00
	*Cynosurus echinatus*	WG	2.77	0.00	1.02	1.25
	*Dactylis glomerata*	WG	0.14	0.58	4.54	4.35
	*Dasypyrum villosum*	WG	6.38	0.00	3.51	0.07
	*Elymus repens*	WG	0.97	0.00	0.00	0.00
	*Gastridium ventricosum*	WG	5.27	0.00	0.00	0.00
	*Holcus lanatus*	WG	0.00	0.00	0.79	0.00
	*Hordeum bulbosum*	WG	0.00	0.00	0.11	0.00
	*Lagurus ovatus*	WG	0.28	0.00	0.00	0.00
	*Lolium arundinaceum*	WG	0.00	0.00	0.00	3.76
	*Lolium perenne*	WG	5.41	0.00	0.00	0.00
	*Melica ciliata*	WG	0.00	0.00	0.00	0.07
	*Oloptum thomasii*	WG	0.83	0.00	0.00	0.00
	*Phalaris minor*	WG	1.11	0.00	0.00	0.00
	*Phleum pratense*	WG	0.14	0.00	0.00	0.00
	*Poa trivialis*	WG	0.83	0.00	0.00	1.71
	*Triticum vagans*	WG	0.28	0.00	0.00	0.00
			49.10	1.68	19.84	15.69
Polygonaceae	*Polygonum aviculare*	WF	0.28	0.00	0.00	0.00
	*Rumex bucephalophorus*	WF	0.00	0.58	0.57	0.00
	*Rumex conglomeratus*	WF	0.14	0.53	0.57	0.00
	*Rumex crispus*	WF	0.00	0.48	0.00	0.00
	*Rumex obtusifolius*	WF	0.00	0.00	0.11	0.00
			0.42	1.59	1.25	0.00
Primulaceae	*Anagallis arvensis*	WF	0.14	0.00	0.00	0.00
	*Cyclamen repandum*	WF	0.00	0.00	0.79	0.00
			0.14	0.00	0.79	0.00
Ranunculaceae	*Clemantis flammula*	EWP	0.00	2.17	0.11	4.02
	*Ranunculus bulbosus*	WF	0.00	0.96	0.00	0.00
	*Ranunculus ficaria*	WF	0.00	0.53	0.00	0.00
	*Ranunculus flammula*	WF	0.00	0.00	0.11	0.00
	*Ranunculus lanuginosus*	WF	0.00	1.25	0.00	0.00
	*Ranunculus repens*	WF	0.00	0.00	2.95	0.07
			0.00	4.91	3.17	4.09
Rhamnaceae	*Rhamnus alaternus*	EWP	0.83	1.35	0.00	0.00
			0.83	1.35	0.00	0.00
Rosaceae	*Crateaegus monogyna*	DWP	0.83	2.60	1.47	9.23
	*Filipendula ulmaria*	WF	0.00	0.34	0.00	0.00
	*Potentilla reptans*	WF	0.00	0.72	0.00	0.00
	*Prunus spinosa*	DWP	0.42	4.57	1.02	15.16
	*Pyrus communis*	DWP	0.00	0.00	0.00	7.78
	*Rosa sempervirens*	EWP	0.00	0.87	1.02	0.66
	*Rubus canescens*	DWP	0.00	1.20	0.00	0.00
	*Rubus* spp.	DWP	4.02	0.00	0.00	0.59
	*Rubus ulmifolius*	DWP	0.00	1.25	5.56	0.00
			5.27	11.55	9.07	33.42
Rubiaceae	*Galium aparine*	WF	0.00	0.00	1.25	0.86
	*Galium cruciata*	WF	0.00	0.00	0.23	0.00
	*Galium verum*	WF	0.00	2.26	0.00	0.00
	*Rubia peregrina*	WF	0.14	10.11	2.38	4.48
	*Sherardia arvensis*	WF	0.00	2.21	0.00	0.00
			0.14	14.58	3.85	5.34
Santalaceae	*Osyris alba*	EWP	0.00	7.51	1.59	0.26
			0.00	7.51	1.59	0.26
Sapindaceae	*Acer campestre*	DWP	0.00	0.00	0.00	0.73
	*Acer monspessulanus*	DWP	0.00	2.36	0.00	0.00
			0.00	2.36	0.00	0.73
Scrophulariaceae	*Verbascum blattaria*	WF	0.00	0.53	0.00	0.00
	*Verbascum sinuatum*	WF	0.00	0.43	0.00	0.00
			0.00	0.96	0.00	0.00
Smilacaceae	*Smilax aspera*	EWP	1.66	0.29	0.23	5.14
			1.66	0.29	0.23	5.14
Solanaceae	*Solanum nigrum*	WF	0.00	0.00	1.70	0.00
			0.00	0.00	1.70	0.00
Ulmaceae	*Ulmus minor*	DWP	0.00	0.58	0.00	0.00
			0.00	0.58	0.00	0.00
Urticaceae	*Urtica dioica*	WF	0.00	0.00	0.23	0.00
			0.00	0.00	0.23	0.00

(^1^) EWP = Evergreen woody plants; DWP = Deciduous woody plants; HWP = Half-woody plants; WF = Wild forbs; WG = Wild graminoids.
